# Tight glycaemic control: a prospective observational study of a computerised decision-supported intensive insulin therapy protocol

**DOI:** 10.1186/cc5964

**Published:** 2007-07-10

**Authors:** Rob Shulman, Simon J Finney, Caoimhe O'Sullivan, Paul A Glynne, Russell Greene

**Affiliations:** 1 Pharmacy Department, University College London Hospitals NHS Trust, 235 Euston Road, London, NW1 2BU, UK; 2 Adult Intensive Care Unit, Royal Brompton Hospital, Sydney Street, London, SW3 6NP, UK; 3 Medical Statistics Group, Joint University College London Hospitals/University College London Biomedical Research Unit, 149 Tottenham Court Road, London, W1P 9LL, UK; 4 Critical Care Department, University College London Hospitals NHS Trust, 235 Euston Road, London, NW1 2BU, UK; 5 Pharmacy Department, School of Health and Biomedical Sciences, Kings College London, 150 Stamford Street, London, SR1 9NH, UK

## Abstract

**Introduction:**

A single centre has reported that implementation of an intensive insulin protocol, aiming for tight glycaemic control (blood glucose 4.4 to 6.1 mmol/l), resulted in significant reduction in mortality in longer stay medical and surgical critically ill patients. Our aim was to determine the degree to which tight glycaemic control can be maintained using an intensive insulin therapy protocol with computerized decision support and to identify factors that may be associated with the degree of control.

**Methods:**

At a general adult 22-bed intensive care unit, we implemented an intensive insulin therapy protocol in mechanically ventilated patients, aiming for a target glucose range of 4.4 to 6.1 mmol/l. The protocol was integrated into the computerized information management system by way of a decision support program. The time spent in each predefined blood glucose band was estimated, assuming a linear trend between measurements.

**Results:**

Fifty consecutive patients were investigated, involving analysis of 7,209 blood glucose samples, over 9,214 hours. The target tight glycaemic control band (4.4 to 6.1 mmol/l) was achieved for a median of 23.1% of the time that patients were receiving intensive insulin therapy. Nearly half of the time (median 48.5%), blood glucose was within the band 6.2 to 7.99 mmol/l. Univariate analysis revealed that body mass index (BMI), Acute Physiology and Chronic Health Evaluation (APACHE) II score and previous diabetes each explained approximately 10% of the variability in tight glycaemic control. BMI and APACHE II score explained most (27%) of the variability in tight glycaemic control in the multivariate analysis, after adjusting for age and previous diabetes.

**Conclusion:**

Use of the computerized decision supported intensive insulin therapy protocol did result in achievement of tight glycaemic control for a substantial percentage of each patient's stay, although it did deliver 'normoglycaemia' (4.4 to about 8 mmol/l) for nearly 75% of the time. Tight glycaemic control was difficult to achieve in critically ill patients using this protocol. More sophisticated methods such as continuous blood glucose monitoring with automated insulin and glucose infusion adjustment may be a more effective way to achieve tight glycaemic control. Glycaemia in patients with high BMI and APACHE II scores may be more difficult to control using intensive insulin therapy protocols. Trial registration number 05/Q0505/1.

## Introduction

In a landmark study [1] of 1,548 patients, the majority of whom had undergone cardiac surgery, intensive insulin therapy (IIT) aiming at achieving tight glycaemic control (TGC) reduced absolute mortality on the intensive care unit from 8% to 4.6%. Patients receiving IIT were managed to an intended target blood glucose of 4.4 to 6.1 mmol/l, whereas control patients were managed to a 'conventional' target blood glucose of 10 to 11.1 mmol/L (conventional insulin therapy [CIT]). The study reported that the benefits of IIT were most pronounced in patients staying more than 5 days in the intensive care unit (ICU). A subsequent reanalysis suggested that 3 days or more were required for benefit to be realized [2]. Furthermore, bloodstream infections, acute renal failure requiring renal replacement therapy, red blood cell transfusions and critical illness polyneuropathy were all reduced in the IIT group. In a subsequent study of 1,200 patients, the same investigators reported that IIT also reduced morbidity in patients admitted to a medical ICU; mortality benefits were only seen in patients treated with IIT for 3 days or longer [2]. Although the overall hospital mortality was no different between the two groups (37% in the IIT group versus 40% in the CIT group), in the longer stay patients mortality was reduced with IIT (53% in the CIT group versus 43% in the IIT group). The reason for the worse outcome with IIT in the shorter stay patients is unclear but it may have been due to the inappropriate inclusion in the study of patients in whom treatment was futile.

Controversy surrounds several areas of TGC. First, the precise blood glucose targets are unclear [3-5]. Data from one observational study [6] suggested that a less stringent target blood glucose range of 4 to 8 mmol/l may achieve similar mortality benefits. Similarly, in a historical single-centre observational study, Krinsley [7] reported a significant reduction in mortality in a mixed medical-surgical ICU following the introduction of an IIT protocol, despite a less stringent blood glucose target of less than 7.8 mmol/l. In contrast, a *post hoc *analysis of the original Leuven study [8] indicated that intermediate glycaemic control, with blood glucose between 6.1 and 8.3 mmol/l, only conferred intermediate advantages when compared with a target range of 4.4 to 6.1 mmol/l.

Second, complex protocols are required to achieve TGC in clinical practice, with frequent blood glucose measurements and changes to insulin infusion rates depending on the rate of change of blood glucose levels [1,7,9]. A major concern about stringent glycaemic targets outside the focus of a clinical trial is that patients may be at increased risk for hypoglycaemia. Several IIT protocols have resulted in significant increases in the incidence of severe hypoglycaemia [1,2,10]. Indeed, this led, at least in part, to the premature cessation of the multicenter German Efficacy of Volume Substitution and Insulin Therapy in Severe Sepsis (VISEP) study [10]. Another protocol was associated with a doubling in the incidence of mild hypoglycaemia (2.3 to 2.8 mmol/l), although severe hypoglycaemic episodes (<2.3 mmol/l) were not more common [7]. A nurse managed IIT protocol (4.5 to 6.1 mmol/l) was associated with significantly fewer hypoglycaemic episodes than a protocol in which insulin dose was adjusted at the attending clinician's discretion [9].

Finally, the existing literature demonstrates that noncomputerized 'paper-based' TGC protocols may not achieve prolonged target glycaemia. The degree of glucose control achieved is not comprehensively described in the Leuven study [1]; the proportion of the time during which the patients' blood glucose concentrations were in the target range was not reported, and blood glucose levels at 06:00 hours were 5.7 ± 1.1 mmol/l in the IIT group, suggesting that about 35.6% of the measurements at this time exceeded 6.1 mmol/l. Results with another paper-based TGC protocol indicated that 58% of samples from 128 patients had a blood glucose concentration in excess of 6.1 mmol/L [11]. Relatively short durations of achievement of target glycaemia were reported following the introduction of a nurse implemented IIT protocol [9]; the duration of time spent within the target glycaemic range was only 11.5 hours/day. In a recent advance, researchers from New Zealand developed and piloted a model-based approach that manages TGC on the basis of controlling nutritional intake in addition to insulin [12,13]. In a pilot study of 19 patients, they reported that 62% of measurements (taken every 1 to 2 hours) were in the glycaemic range from 4.1 to 6.1 mmol/l in a general ICU population.

We hypothesized that implementing an IIT protocol (Additional file 1) using computerized decision support would reduce the incidence of hypoglycaemia and increase the proportion of time spent within the target glycemic range. Therefore, we implemented a modified IIT protocol [9] into a bedside clinical information system. The protocol was aimed at achieving a blood glucose of 4.4 to 6.1 mmol/l. We considered that the computerized decision support would make a complex IIT protocol feasible in a busy clinical setting.

The aims of this study were to evaluate the quality of glucose control achieved by this system and to identify any factors that may explain variability in glycaemic control.

## Materials and methods

### Patients

This was an observational study of patients treated using the IIT protocol on the ICU. Following ethical approval and training of medical and nursing staff, consecutive patients admitted to the Middlesex and University College Hospitals ICU who were treated with the IIT protocol, from 10 January to 25 June 2005, were studied prospectively. Patients were included if they were mechanically ventilated, and it was anticipated that this would continue for at least 24 hours. Additional inclusion criteria included the presence of a central venous line and arterial cannula. Patients with diabetic ketoacidosis or diabetic nonketotic hyperosmolar coma were excluded.

### Intensive insulin therapy protocol

The IIT protocol (Additional file 1) was developed by the ICU consultant (PG), based on a published protocol [9], in conjunction with the senior ICU team including doctors, nurses and the unit pharmacist (RS). The protocol was introduced after a comprehensive educational programme into the rationale and logistics of glucose control in the critically ill. The protocol was facilitated by a computerized decision-support system that was designed in-house as part of our clinical information system (QS; GE Healthcare, Anapolis, MD, USA). The protocol was initiated 2 months before the study started in order to allow time to resolve initial difficulties. In this computerized decision support system, the nurse inputs the blood glucose measurement and the current insulin dose into the bedside computer. The decision support system uses this blood glucose value and the previous measurement to derive a new recommended insulin dose, following the IIT protocol (Additional file 1). For example, if the insulin was currently running at 6 units per hour and the latest blood glucose measurement was 4.4 mmol/l, whereas the previous blood glucose measurement was 6 mmol/l, then the program would recommend reducing the insulin dose to 5 units per hour.

The ICU employs an early enteral feeding strategy, and feeding is usually commenced within 24 hours of admission. Feeding is initiated at 30 ml/hour, and this is doubled if it is adequately tolerated after 4 hours. If tolerated, after a further 4 hours the feed is optimized to a final rate in relation to the patient's weight. The IIT protocol dictated that glucose 50% infusion was administered until full enteral feeding was established. The glucose was administered at a constant rate from accurate volumetric infusion devices.

Intravenous corticosteroids were administered as intermittent boluses, rather than as a continuous infusion. No attempt was made to avoid medications diluted in glucose and administered intermittently. Continuous intravenous infusions were routinely diluted in 5% glucose, unless there were specific incompatibilities.

### Data collection

Blood glucose data were obtained from two sources: glucose meter readings (Glucometer Elite™; Bayer Diagnostics, Newbury, UK) and the ICU blood gas analyzer (ABL 625; Radiometer, Crawley, UK). The blood gas analyzer underwent daily control by the unit's medical physicists. After 1 month glucose meters were also used to guide IIT to facilitate bedside management. Only blood glucose levels measured from arterial blood samples (not finger sticks) were used to guide therapy in the IIT protocol. All baseline, outcome data and concomitant drugs affecting glycaemia were recorded from the clinical information system.

Severe hypoglycaemia (blood glucose ≤2.2 mmol/l) and hyperglycaemic (blood glucose >10 mmol/l for >2 hours) events were individually analyzed to identify features that were probably causative.

### Analysis of glucose control

The blood glucose findings for each patient were manually input into the clinical information system by the bedside nurse, and this was downloaded into a Microsoft Excel spreadsheet. A macro formula was used to calculate the time spent in seven predefined glycaemic bands (0 to 2.2, 2.3 to 4.39, 4.4 to 6.1, 6.2 to 7.99, 8 to 9.9, 10 to 11.1, and >11.1 mmol/l). It was assumed that blood glucose values trended linearly between successive measurements. An element of protocol adherence was studied by assessment of whether each blood glucose assay was conducted within the time stated in the protocol. Although it would be expected that the assays would follow the time interval stated in the protocol, it was recognized that there might be a delay until the nurses recorded the result on the computer, and hence a 50% tolerance limit was accepted in the assessment of protocol adherence.

Analysis of the impact of drugs affecting glycaemic control was undertaken by recording those who were prescribed these drugs for a part or the whole of their IIT course, and comparing glycaemic control between these patients and those who were not prescribed these agents. Commonly used drugs known to affect blood glucose levels are listed in Table 1.

**Table 1:** Commonly used medications that can produce hypoglycaemia and hyperglycaemia as adverse effects

Adverse effect	Drugs
Hypoglycaemia	Angiotensin-converting enzyme inhibitors
	Budesonide
	Chlorpromazine
	Disopyramide (isolated cases)
	Ethanol
	Quinine
Hyperglycaemia	Adrenaline (ephinephrine)
	β_2 _agonists (in diabetes)
	Ciclosporin
	Clonidine
	Corticosteroids
	Diazoxide
	Diuretics (mainly thiazides)
	Glucose
	Isoniazid
	Nicotinic acid
	Noradrenaline (norephinephrine)
	Octreotide
	Olanzapine
	Oral contraceptives
	Phenytoin
	Risperidone
	Rituximab
	Theophylline
Miscellaneous	Acetazolamide (potentiates action of hypoglycaemics)
	Amitriptyline (elevates or decreases blood sugar levels)
	Imipramine (isolated cases of increase or decrease in blood sugar levels)
	Pentamidine (life threatening hypoglycaemia, less severe hyperglycaemia)
	Tacrolimus (elevates or decreases blood sugar levels)
	Triamterene (impaired glucose metabolism [<1/100] [33])

### Statistical analysis

All data analyses were conducted using SPSS 13.0 for Windows (SPSS Inc., Chicago, IL, USA). Data are presented as mean ± standard deviation or as median (interquartile range), as appropriate. Variables thought potentially to be clinically relevant to TGC were identified *a priori*. Linear regression was used to help identify factors that were associated with TGC.

## Results

Fifty consecutive ICU patients were recruited, and their baseline characteristics are summarized in Tables 2 and 3 and outcomes in Table 4.

**Table 2:** Baseline characteristics of patients

Characteristic	Value
Male (*n *[%])	34 (68%)
Age (years; median [IQR])	66 (54 to 73)
BMI (median [IQR])^a^	25.5 (22.3 to 29.1)
Underweight (BMI <18.5 kg/m^2^; n [%])	4 (8%)
Overweight but not obese (BMI 25 to 30 kg/m^2^; *n *[%])	19 (38%)
Obese (BMI >30 kg/m^2^; *n *[%])	6 (12%)
Patients taking drugs influencing glycaemia (*n *[%])^b^	36 (72%)
Parenteral nutrition for all or part of admission (*n *[%])	9 (18%)
Enteral nutrition for all or part of admission (*n *[%])	43 (86%)
Glucose 50% infusion for all or part of admission (*n *[%])	42 (84%)
APACHE II score (first 24 hours; median [IQR])^c^	23 (17–29)
SAPS II score (first 24 hours; median [IQR])^d^	47 (35–64)
History of diabetes (*n *[%])	6 (12%)
Treated with insulin (*n *[%])	1 (2%)
Treated with oral agents (*n *[%])	4 (8%)
Diet controlled (*n *[%])	2 (4%)

Fifty patients were included in the study. ^a^Body mass index (BMI) calculated as weight (kg) divided by height (metres) squared. ^b^See Table 1 for a list of commonly used drugs known to affect glycaemia. ^c^Acute Physiology and Chronic Health Evaluation (APACHE) II score range 0 to 71. ^d^Simplified Acute Physiology Score (SAPS)II range 31 to 163. IQR, interquartile range.

**Table 3:** Reason for intensive care

Reason for intensive care	All patients^a^	Surgical^a^	Medical^a^
Cardiac	5 (10%)	4 (21.1%)	1 (3.2%)
Abdominal	6 (12%)	5 (26.3%)	1 (3.2%)
Trauma	2 (4%)	0 (0%)	2 (6.5%)
Sepsis	3 (6%)	1 (5.3%)	2 (6.5%)
Respiratory failure	23 (46%)	7 (36.8%)	16 (51.6%)
Liver failure	4 (8%)	1 (5.3%)	3 (9.7%)
Pancreatitis	1 (2%)	0 (0%)	1 (3.2%)
History of malignancy	16 (32%)	5 (26.3%)	11 (35.5%)
Total	50 (100%)	19 (38.0%)	31 (62.0%)

Fifty patients were included in the study. Values are expressed as number (%). ^a^Patients could have more than one reason for intensive care.

**Table 4:** Patient outcomes

Outcome	Value
ICU LOS (days; median [IQR])	7.0 (3.0 to 21.3)
28-day survival (*n *[%])	34 (64.2%)
Mechanical ventilation (days; median [IQR])	5.5 (2.0 to 15.3)
Patients haemofiltered (*n *[%])	13 (26.0%)
Days on haemofiltration (median [IQR])^a^	4.0 (1.5 to 7.5)

Fifty patients were included in the study. ^a^For those patients undergoing haemofiltration. ICU, intensive care unit; IQR, interquartile range; LOS, length of stay.

The median (interquartile range) duration of the IIT course was 4.3 (1.4 to 11.5) days. Eight (16%) patients had an IIT course of less than 24 hours. Twenty-two patients (44%) had an IIT course of less than 3 days. A total of 7,209 blood glucose measurements (including glucose meter and blood gas analysis) were recorded for the 50 patients, over a total time of 9,214 hours. The number of assays taken specifically to guide IIT was 4,891, equating to one measurement every 113 min.

**Figure 1:**
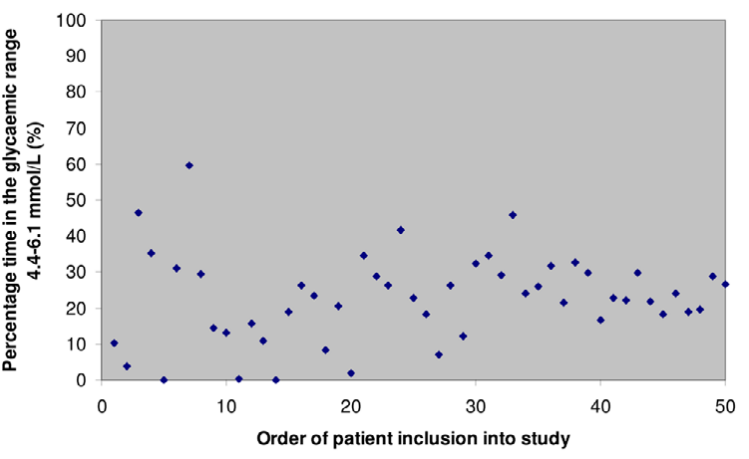
Percentage of time spent in target glycaemic range for each patient, in order of inclusion in study.

The median (interquartile range) time taken from the initiation of the IIT protocol to first achievement of the target range of 4.4 to 6.1 mmol/L was 10.5 (4.8 to 14.5) hours. Graphical exploration demonstrated that the proportion of time spent in the target blood glucose range did not change over the 6-month study period (Figure 1).

**Figure 2:**
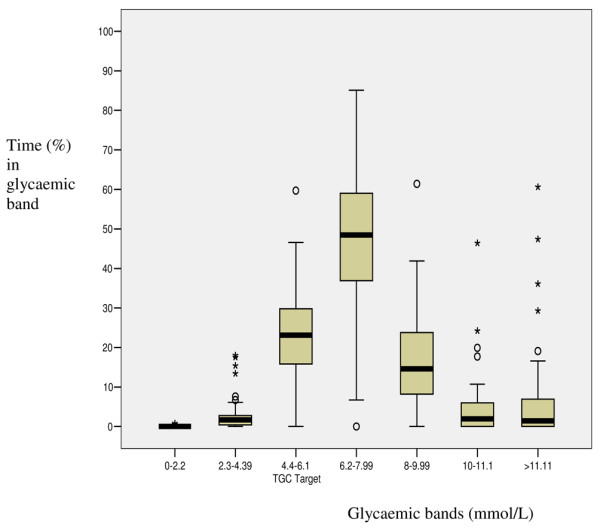
Box and whisker plot of percentage time in predefined glycaemic ranges. TGC, tight glycaemic control.

The time spent in each glycaemic band was determined and expressed as a percentage of the total duration of IIT (Figure 2). Patients spent the most time (median 48.5%) with a blood glucose between 6.2 and 7.99 mmol/l. The TGC target of 4.4 to 6.1 mmol/l was achieved for a median of 23.1% of the therapy. Relatively brief proportions of time were spent in the hyperglycaemic ranges 10 to 11.1 mmol/l and above 11.11 mmol/l (median 2.0% and 1.4%, respectively). The degrees of glycaemic control for various patient groups are presented in Table 5.

**Table 5:** Glycaemic control of various patient groups

Patient group	Blood glucose	
	4.4 to 6.1 mmol/l	6.2 to 7.99 mmol/l
All patients (*n *= 50)	23.1% (15.4 to 29.8)	48.5% (36.9 to 60.8)
Surgical patients (*n *= 19)	23.9% (14.4 to 31.0)	55.7% (40.6 to 63.9)
Medical patients (*n *= 31)	22.1% (16.7 to 29.8)	40.3% (30.0 to 56.6)
History of diabetes (*n *= 6)	11.6% (2.9 to 20.8)	36.3% (15.3 to 43.5)
No history of diabetes (*n *= 44)	23.9% (18.4 to 30.7)	51.6% (38.8 to 63.0)
Co-prescription of drug(s) causing hyperglycaemia (*n *= 32)	26.3% (19.0 to 30.7)	47.7% (37.8 to 59.7)
Co-prescription of drug(s) causing hypoglycaemia (*n *= 5)	7.0% (2.0 to 31.6)	62.8% (17.4 to 75.4)

Shown are the percentages of the intensive insulin therapy course spent in the specified glycaemic ranges. Values are expressed as median (interquartile range).

Hypoglycaemic events were defined as episodes during which the blood glucose was 2.2 mmol/l or less. This occurred on 14 occasions, affecting five patients (10%). These included one medical and four surgical patients. No clinical sequelae were noted from these episodes. Cumulatively, little time (median 0.04%) was spent in the severely hypoglycaemic range of 0 to 2.2 mmol/l or in the hypoglycaemic range 2.3 to 4.39 mmol/l (median of 1.7%). Some common themes emerged. First, the events did not appear to be associated with the effects of additional medication, although one of the patients did receive a corticosteroid at the time of the event. In several cases the protocol was not followed. It was common for blood glucose samples not to have been taken frequently enough. Interruption in enteral feed was another common cause of hypoglycaemia. In three instances the records on the information system gave conflicting and ambiguous information, making interpretation impossible and suggesting inadequate documentation.

There were 28 hyperglycaemic episodes (defined as blood glucose >10 mmol/l for at least 2 hours). Fifteen (30.0%) patients treated with the IIT protocol experienced at least one hyperglycaemic episode. Of the 28 episodes, 19 (68%) occurred within the first 36 hours of therapy and appeared to correspond to nasogastric feeding plus glucose supplementation.

Box-plots (not shown) comparing glycaemic profiles of patients administered medicines causing hyperglycaemia (*n *= 32) with the profiles of those who were not receiving such medications (*n *= 18) did not show any clear difference. This may reflect the relatively small number of cases, however.

It was found that a median 47.0% (32.9% to 59.0%) of assays were not taken within the time frame stated in the IIT protocol. However, in the univariate analysis, the proportion of correctly timed assays did not account for the variability in the percentage of time spent within the target TGC range (Table 6).

**Table 6:** Univariate analysis of factors affecting the percentage time in the target glycaemic range

Parameter	*P*	R^2^
BMI	0.01	13.1%
APACHE II score	0.02	10.8%
History of diabetes mellitus	0.02	10.2%
Gender	0.06	7.1%
Drugs affecting glycaemia	0.27	2.5%
Percentage of correctly timed assays	0.63	0.5%
Age	0.75	0.2%

APACHE, Acute Physiology and Chronic Health Evaluation; BMI, body mass index.

In this study, the percentage time spent in the target range (blood glucose 4.4 to 6.1 mmol/l) was taken to describe TGC, and the distribution was found to satisfy the conditions of a normal distribution. Seven factors were chosen that might clinically be expected to influence TGC (Table 6). Univariate analysis (Table 6) suggested that body mass index (BMI) accounted for 13% of the variability, with a higher BMI associated with a lower TGC. Acute Physiology and Chronic Health Evaluation (APACHE) II score accounted for 11% of the variability, again with a higher score associated with poorer glycaemic control. TGC was worse in patients with diabetes mellitus (10% of the variability explained). There was a suggestion that females had better TGC than males, by an average of 7.1%. Age did not appear to explain variability in TGC.

In the multivariate analysis (Table 7), BMI, sex, previous diabetes and APACHE II score accounted for 27% of the variability in percentage time spent in the target TGC range.

**Table 7:** Multivariate analysis of factors affecting the percentage time in the target glycaemic range

Parameter	*P*	R^2^
BMI	0.04	
APACHE II score	0.04	
Sex	0.19	
Previous diabetes	0.50	
		27%

APACHE, Acute Physiology and Chronic Health Evaluation; BMI, body mass index.

## Discussion

The key finding was that attempting to achieve TGC, using a complex protocol assisted by computerized decision support, was extremely difficult. Our experience highlights the difficulties of applying the results of highly controlled clinical trials to everyday practice [14]. The target blood glucose range of 4.4 to 6.1 mmol/l was only achieved for a median of 23% of the time that the protocol was used, although the next band – 6.2 to 7.99 mmol/l – was achieved for 49% of the time. The time to first reach the target blood glucose range of 10.5 hours was comparable to that reported by Kanji and co-workers [9] with a similar target.

This main finding has not been a feature of other published work. It contrasts starkly with the findings reported by Kanji and coworkers [9], in which the similar target range of 4.5 to 6.1 mmol/l was achieved for 47% of the time in the protocol group. It is also discordant with the report from the Specialized Relative Insulin and Nutrition Tables (SPRINT) investigators [12], who found that over 60% of the results were in the 4.1 to 6.1 mmol/l. Dutch investigators developed and evaluated the Glucose Regulation for Intensive care Patients (GRIP) computerized decision support system in a short-stay cardiac ICU, aiming for a target blood glucose of 4 to 7.5 mmol/L [15]. This target was achieved for 78% of the time, following a median of 4.9 daily blood glucose assays. The Glucommander insulin dosing software has been in use for many years, and good glycaemic control has been reported in mixed patient groups; however, evidence in critically ill patients has not been reported separately [16]. In both Leuven studies [1,2] there was insufficient detail in the results to allow adequate assessment of the degree of control achieved in terms of the proportion of course in various glycaemic ranges. Other studies reporting on similar aspects of control did not employ the key aspects of the Leuven study, namely a blood glucose target of 4.4 to 6.1 mmol/l and the initial glucose load [15,17-22].

There is a clear need to standardise the protocols for IIT, in order to address differences in glycaemic targets, nutritional and glucose supplementation, and insulin dosing strategy. Our experience suggests that many of the hyperglycaemic events occurred during the first 36 hours of IIT, which coincides with the establishment of enteral feed while intravenous glucose is concomitantly administered. This glucose infusion was solely part of the IIT protocol and was not administered to other ICU patients prescribed insulin. The clinical value of this initial glucose infusion is not known and would depend on whether IIT works by reducing hyperglycaemia or through another action of insulin, which may be anti-inflammatory, anti-coagulant, or anabolic [23]. Our protocol has now been amended to remove the glucose supplementation as we employ early enteral feeding. This change has also obviated the need for a central line, which was required for the glucose 50% infusion. Removal of glucose supplementation is in keeping with practice in some other units [24]. An alternative would have been to adopt a dynamic glucose algorithm that includes the total glucose (including any feed) as an input. A dynamic algorithm uses data from a previous blood glucose response to a change in insulin, and thus it 'learns' how an individual patient responds to insulin and feed, and advises accordingly. Other protocol changes include employing IIT only in those patients who the admitting doctor expects to be on the ICU for at least 3 days. Furthermore, we have now employed a simplified frequency of blood glucose measurement (to address the low frequency of blood glucose assay rate found in this study) and an educational program to enforce the importance of adherence. The impact of these changes will be analyzed in due course.

There are differences in the IIT protocols in use [24]. Research is required to identify the most effective protocol and the best way to organize the ICU management of IIT [24,25]. The incidence of severe hypoglycaemia was too low and our study was insufficiently powered to assess reliably any association with survival. Because this was a study of existing practice, no *a priori *acceptable levels of efficacy or safety were set.

The method used here to estimate the time during which the blood glucose concentration was within the predefined glycaemic bands [6] was superior to those used in other studies, in which results were pooled and the mean reported [17,18,21,22]. In another study with a target blood glucose of 4.1 to 6.1 mmol/l [12], the findings were presented as the proportion of results within this range and a wider range. In another study of true TGC (aiming for a blood glucose concentration of 4.4 to 6.1 mmol/l) [9], an assumption was made that the time between two measurements reflected the time spent at the later measurement; for instance, if a blood glucose reading was 5 mmol/l, then the time recorded within the target range was the interval between this and the immediately preceding measurement. This assumption is difficult to justify. The method used to describe glycaemic control in our study is recommended if blood glucose is not measured at a uniform frequency.

The factors identified that may explain variability in TGC were BMI, APACHE II score, previous diabetes and sex. As a practical consequence, patients with high BMI or APACHE II scores may require more frequent blood glucose measurement if IIT is employed. Because APACHE II score is a severity of illness scale that correlates well with mortality [26], it may be that a high score reflects critical illness and associated insulin resistance. Finney and co-workers [6] identified an association between increasing insulin dose and increased mortality; this was also reported by Van den Berghe and co-workers [8].

The finding that 16% of patients had an IIT course of under 24 hours suggests that they were not expected to have the mortality gains from IIT reported in the major studies [1,2]. The appropriate reasons for these short courses were futility of care (two cases) and conversion from IIT to a glucose-insulin-potassium regimen (one case). The inappropriate reason for a short IIT course was on extubation with a short course of mechanical ventilation (five cases). This highlights the difficulty in predicting which patients will have longer stays in the ICU [2]. However, the blood glucose data from these patients were included in the results because we aimed to describe implementation of IIT in 'real life'.

The finding that 47% of blood glucose assays were not within the time frame stated in the protocol may reflect burden of additional work for the nurse that is associated with IIT. Interestingly, however, there did not appear to be a relationship between correct timing of blood glucose assays and TGC, based on the univariate analysis. As in most areas of therapy, collaboration of the nurses is crucial to successful application of IIT. The significant impact of IIT on nursing workload is such that nurses should be involved in all aspects of protocol design, training, implementation and revaluation. Our protocol has subsequently been amended, in the light of these findings and in discussion with the nurses, to reduce the frequency of blood glucose monitoring to a more reasonable frequency (usually 2 hourly, rather than hourly).

Computerized clinical decision support has been defined as information systems designed to improve decision making, including patient-specific advice, which is reviewed by a health care practitioner before clinical action [27]. Our system met these criteria. One view is that it is not the paper or computer that makes the difference in how well TGC is achieved; rather, it is the protocol used. TGC was suitable for computerization because the IIT protocol was complex and would have been time consuming for nursing staff to use had it not been for the computerized decision support.

The main limitation of the study was that blood glucose analysis included measurements based on two different instruments: blood gas analyzer and glucose meter. However, when the analysis was repeated only using the blood glucose results used by the nurses (predominantly glucose meter) for the decision support, similar results were observed; indeed, this would have been more of an issue had we employed capillary blood (fingerstick) measurement [28], which we did not. It is acknowledged that the quality control of bedside glucose meters was not conducted regularly in a uniform manner. We were unable to record the amount of insulin administered. Finally, we did not formally investigate the staff perception of the IIT protocol.

## Conclusion

The introduction of a computerized decision supported IIT protocol did not produce high quality glycaemic control. Hyperglycaemia was particularly prevalent during the early stages of IIT, when enteral feed was being established while exogenous glucose was being administered. Whether exogenous glucose is required at the onset of IIT merits further investigation. Futhermore, poorer glycaemic control occurred in those patients with a higher BMI and APACHE II score. More flexible protocols may be required for these patients. Indeed, intelligent closed loop systems that adjust insulin based on previous responses are being developed [29,30]. Coupled with continuous intravenous glucose sensors [31,32], these may be able to provide near perfect control.

## Key messages

• TGC was not achieved to a large extent despite a computerized decision supported IIT protocol.

• Glycaemia was in the range 4.4 to 7.99 mmol/L for approximately 75% of the time.

• Hyperglycaemia-causing drugs did not appear to influence glycaemic control.

• BMI, APACHE II score and diabetes each explained approximately 10% of the variability on univariate analysis.

• BMI and APACHE II score explained 27% of the variability in multivariate analysis.

## Abbreviations

APACHE = Acute Physiology and Chronic Health Evaluation; BMI = body mass index; CIT = conventional insulin therapy; ICU = intensive care unit; IIT = intensive insulin therapy; TGC = tight glycaemic control.

## Additional files 

Additional file 1 is a pdf file summarizing the ICU intensive insulin therapy protocol, 2004 to 2006.  

## Competing interests

The authors declare that they have no competing interests.

## Authors' contributions

RS was responsible for conception and design of the study, data collection and analysis and interpretation of results, and drafted the manuscript. CO advised on the design, statistical analysis and the draft. SJF advised on study design, analysis, interpretation and the draft. PAG and RG advised on conception, design and interpretation. RG advised on the draft. All authors approved the final version of the manuscript to be published.

## Supplementary Material

Additional file 1 A pdf file summarizing the ICU intensive insulin therapy protocol, 2004 to 2006. Click here for file
