# Supplementary material for: Tight glycaemic control: a prospective observational study of a computerised decision-supported intensive insulin therapy protocol
Source: Crit Care. 2007 Jul 10;11(4):R75. doi: 10.1186/cc5964 (PMC2206495; doi:10.1186/cc5964)
Supplement: Additional file 1 — A pdf file summarizing the ICU intensive insulin therapy protocol, 2004 to 2006. [file cc5964-S1.pdf]

All eligible patients **must** be within **24 hours** of admission ICU and likely to be ventilated for >24 hours. Diabetic emergencies (diabetic ketoacidosis; non-ketotic, hyperosmolar coma) are not suitable. Only measure blood glucose using a glucometer, not the gas machine.

Feeding or glucose

Run IV 50% glucose at **20 ml/hour** centrally until **full** enteral feeding or TPN is established. Full enteral feeding means feed is running at full target rate with minimal aspirates (usually after 4 hours, as per ICU feeding algorithm). Once **full** feeding established, wean 50% glucose infusion by 5 ml/h every hour until off.

Initial infusion rate

**First blood glucose (mmol/l)**	< 6.1	6.1-8	8.1-12	> 12.0
**Starting insulin infusion rate (units/h)**	0	1	2	3

Insulin-dependent diabetics should always have a minimum insulin infusion rate of ≥ 0.5 u/h.

Subsequent insulin dosing

**Blood glucose (mmol/l)**	**Change rate of insulin infusion (units/h)**
< 2.5	STOP INSULIN INFUSION. GIVE 50 ML 50% GLUCOSE; recheck BG every 15 min. After 1 h, recommence insulin according to the table in Section B.
2.5 – 3.9	STOP INSULIN INFUSION. GIVE 25 ML 50% GLUCOSE; recheck BG every 15 min. After 1 h, recommence insulin according to the table in Section B.
4 – 4.3	Reduce dose by 50%; measure BG every 15 min until BG >4.4 mmol/l
4.4 – 6.1<br>**Target range**	If BG lower than last measurement – reduce by 1 unit/h<br>If BG lower by more than 50% of last measurement – reduce dose by 50% and check BG every 15 min (see section 2)<br>If BG unchanged or higher than last measurement – no change
6.2 – 7.7	If BG lower than last measurement – no change<br>If BG lower by more than 50% of last measurement – reduce dose by 50% and check BG every 15 min (see section 2)<br>If BG unchanged or higher than last test – increase by 0.5 unit/h
7.8 – 11	If BG lower than last measurement – no change<br>If BG lower by more than 50% of last measurement – reduce dose by 50% and check BG every 15 min (see section 2)<br>If BG unchanged or higher than last test – increase by 1 unit/h
11.1 – 14	If BG lower than last measurement – no change<br>If BG lower by more than 50% of last measurement – reduce dose by 50% and check BG every 15 min (see section 2)<br>If BG unchanged or higher than last test – increase by 2 unit/h
≥ 14.1	Increase rate by 2 units/h. If BG > 14.1 for 3 consecutive tests, increase insulin rate by 50%, check BG every 15 min and call Physician.

How should blood glucose (BG) be monitored?

i. ***Only measure BG using a glucometer***
ii. Check BG hourly until 3 consecutive values within the target range
iii. Check BG 2 hourly thereafter for 24 h. If BG stable & within the target range, measure 4 hrly, providing no significant clinical change, & no change in nutritional intake
iv. If any of the following occur, check BG hourly until 3 consecutive values within the target range:
   - any change in insulin infusion rate
   - significant change in clinical condition
   - initiation or cessation of vasopressor or steroid therapy
   - initiation or cessation of renal replacement therapy
   - initiation, cessation or rate of change of nutritional support
v. If BG < 4.4 mmol/l, measure every 15 min

When to stop intensive insulin therapy protocol?

- ❏ **STOP INSULIN** during any interruption to enteral or parenteral feeding (e.g. patient transfer to CT scan, prior to surgery, aspiration during feeding protocol). If restarting the protocol, insulin should commence at the start of the protocol once feeding re-established, or once 50% glucose infusion recommenced
- ❏ If the patient’s treatment and / or condition necessitates frequent interruptions to the protocol, discuss with Consultant or Nurse-in-Charge about protocol discontinuation
- ❏ Stop protocol 4 hours prior to patient transfer outside ICU, but continue to monitor blood glucose at least hourly
- ❏ Stop protocol if arterial cannula removed
- ❏ Stop protocol and revert to conventional insulin therapy (or preadmission diabetic therapy, if any) 24 h prior to discharge from ICU
- ❏ Do not stop the protocol if patients are extubated, providing they have an arterial cannula *in situ.*

DOCUMENT ALL REASONS FOR DISCONTINUING THE PROTOCOL IN THE NOTES
